# Evaluation of hand hygiene belief and practice among healthcare workers at tertiary hospital, mogadishu, somalia: a cross-sectional study

**DOI:** 10.1038/s41598-025-34843-1

**Published:** 2026-01-09

**Authors:** Figen Balcioğlu, Marian Muse Osman, Tigad Abdisad Ali, Suad Abdikarim Isse, Ahmed Dogan, Ali Kutta Çelik, Fadumo Nur Adan, Fartuun Ahmed Mohmud, Yahye Sheikh Abdulle Hassan

**Affiliations:** 1 Department of Infectious Control Nurse, Mogadishu-Somalia-Turkey Recep Tayyip Erdoğan Training and Research Hospital, Mogadishu, Somalia; 2 Research Department, National Institute of Health, Mogadishu, Somalia; 3 Benadir Institute for Research and Development, Benadir University, Mogadishu, Somalia; 4 Research Department, Royal Hospital, Mogadishu, Somalia; 5 Department of Infectious Diseases and Clinical Microbiology, Mogadishu-Somalia-Turkey Recep Tayyip Erdoğan Training and Research Hospital, Mogadishu, Somalia; 6 Department of Education, Mogadishu-Somalia-TurkeyRecep Tayyip Erdoğan Training and Research Hospital, Mogadishu, Somalia; 7 Faculty of Medicine and Health Sciences, Jamhuriya University of Science and Technology, Mogadishu, Somalia

**Keywords:** Disease prevention, Health services

## Abstract

Healthcare-associated infections (HAIs) pose a significant challenge in resource-limited settings, such as Somalia, where hand hygiene is recognized as a primary preventive measure. This study assessed hand hygiene beliefs and practices among healthcare workers (HCWs) at a tertiary hospital in Mogadishu, Somalia. A cross-sectional study was conducted from April to June 2024 involving 304 randomly selected HCWs at the Mogadishu Somali-Turkiye Recep Tayyip Erdogan Training and Research Hospital. Data was collected using the Hand Hygiene Beliefs Scale (HHBS) and Hand Hygiene Practices Inventory (HHPI). Data were analyzed using descriptive statistics and a Generalized Linear Model (GLM) to identify significant factors associated with belief and practice scores, with significance set at . The findings revealed a strong belief in hand hygiene but only moderate adherence to practices. The multivariable GLM analysis indicated that for hand hygiene beliefs, holding a Master’s degree degree $(\beta=-0.257,p=0.041)$ and having 10–15 years of experience $(\beta=-0.391, p=0.001)$ were significantly associated with lower practice scores. A significant discrepancy exists between hand hygiene beliefs and practices, influenced heavily by educational level, profession, and years of experience. Enhancing patient safety requires targeted, systemic interventions, such as peer-mentoring for nurses, refresher training for mid-career professionals, and specialized monitoring in high-risk departments to bridge the gap between belief and clinical application.

## Introduction

Globally, healthcare-associated infections (HAIs) continue to pose significant health challenges, with recent studies highlighting their substantial impact on patient outcomes and healthcare systems^1^. However, hand hygiene is recognized as a crucial preventive measure against HAIs; healthcare workers’ (HCWs) adherence still needs to be improved, particularly in settings with limited resources^2,3^.

HAIs are a major global issue, with an estimated 10% of patients in low-income nations contracting illnesses due to poor hand hygiene practices^4,5^. Hand hygiene is an effective and essential means to reduce disease transmission through direct contact. HAIs result in prolonged hospitalization, higher mortality rates, and significant financial and personal costs^6^. Thus, enhancing hand hygiene compliance among healthcare workers is vital to reducing these negative consequences^4,5^.

Their beliefs and attitudes significantly influence how healthcare workers follow hand hygiene protocols. Several factors affect hand hygiene practices, including personal perspectives, competence levels, job demands, and accessibility to hand hygiene supplies^7^. Understanding these elements is crucial for developing effective strategies to enhance compliance. Furthermore, healthcare facilities in developing countries, such as Somalia, face additional obstacles such as restricted access to clean water, soap, and alcohol-based hand rubs, which hinder adherence to hand hygiene protocols^8^. Research has revealed that implementing educational programs, providing training, and providing continuous supervision can significantly improve hand hygiene compliance among healthcare workers^9,10^.

Somalia’s healthcare system presents a particularly compelling case study, having been profoundly impacted by decades of conflict and instability. Hospitals in Mogadishu operate under exceptional circumstances, serving a large and vulnerable population^11–13^. The risk of nosocomial infections is heightened by inadequate infection prevention measures, including inadequate hand hygiene protocols^14–16^. However, there is a critical knowledge gap regarding how the hand hygiene beliefs and practices of healthcare workers have evolved in the aftermath of the COVID-19 pandemic, particularly in this challenging context.

This study addresses a crucial knowledge gap by conducting the first comprehensive evaluation of hand hygiene beliefs and practices among healthcare professionals in a Somali tertiary hospital following the COVID-19 pandemic. The study’s significance lies in two key aspects. Firstly, it examines the relationship between healthcare workers’ hand hygiene beliefs and their actual practices, focusing on a resource-constrained, post-conflict setting. Secondly, it uncovers specific obstacles to and enablers of hand hygiene adherence, providing valuable insights that directly address the unique challenges present in this context. These findings will inform the development of targeted, contextually appropriate interventions to enhance patient safety and reduce infection rates in Somalia. Therefore, this study aimed to assess hand hygiene beliefs and practices among healthcare workers at tertiary hospitals in Mogadishu, Somalia.

## Materials and methods

### Study Design, Setting, Period, and population

This cross-sectional study was conducted between April and June 2024 at the Mogadishu Somali Turkiye Recep Tayyip Erdogan Training and Research Hospital, a major tertiary-level referral hospital in Mogadishu, Somalia. This hospital offers a range of healthcare services and has a diverse workforce, making it an ideal setting for evaluating hand hygiene practices and attitudes among healthcare workers (HCWs). The study population comprised all full-time clinical healthcare workers with direct patient contact at the hospital. These cadres included Physicians, Nurses, midwives, pharmacists, and professionals allied to medicine.

## Inclusion and exclusion criteria

All HCWs who provided written informed consent were eligible for inclusion. Administrative staff without patient contact, as well as HCWs who were on leave or otherwise unavailable during the data collection period, were excluded.

## Sample size determination and sampling technique

The sample size was determined using an online calculator^17^, with parameters set at a 95% confidence level, a 5% margin of error, and an assumed 50% prevalence of hand hygiene compliance, as the actual prevalence in this setting was unknown. For a total target population of 1,150 HCWs, the initial required sample size was 289. To account for potential non-responses, the sample size was increased by 5%, resulting in a final target of 304 participants. A stratified random sampling method was employed to ensure sufficient representation of the different cadres of healthcare professionals. Each hospital department was considered a stratum, and participants were randomly chosen from each stratum.

## Data collection tools

This study employed a self-administered questionnaire comprising three primary sections. Demographic characteristics, including age, gender, occupation, experience duration, work schedules, and department, were gathered in the initial section. The second part employed the Hand Hygiene Beliefs Scale (HHBS), created by Thea van de Mortel and later adapted for use in Turkey by Karadağ. This 22-item scale assesses hand hygiene beliefs (19 questions) and perceived importance (3 questions) using a 5-point Likert-type scale ranging from 1 (Strongly Disagree) to 5 (Strongly Agree). Seven items required reverse scoring, and the total score ranged from 22 to 110, with higher scores indicating a more positive attitude toward hand hygiene^18^.

The final section incorporated the Hand Hygiene Practices Inventory (HHPI), comprising 15 questions rated on a 5-point Likert scale. The HHPI scores ranged from 15 to 75, with higher scores indicating superior hand hygiene practices^18,19^ A pilot study involving 20 participants (10% of the study population) was conducted to ensure the reliability of the results. The researcher assessed the questionnaire’s internal consistency using Cronbach’s alpha, yielding acceptable values of 0.72 for the HHBS and 0.80 for the HHPI.

### Data collection procedure

A team of trained research assistants systematically visited all hospital departments, ensuring coverage across different shifts, to obtain a representative sample of healthcare workers. Before the survey administration, potential participants were informed of the study objectives, and those who consented to participate provided written informed consent. The questionnaire was distributed during periods of low activity in the participants’ shifts to minimize disruptions to patient care. Respondents were provided with a designated quiet area and allocated 15–20 min to complete the questionnaire independently. To optimize the response rates and data quality, the researchers remained close to clarifying any inquiries while maintaining participant confidentiality. Completed questionnaires were promptly collected and assessed for their completeness.

## Data management and analysis

Data were entered and cleaned using Microsoft Excel and subsequently analyzed using Stata version 17.0. Descriptive statistics, including frequencies and percentages, were used to summarize the sociodemographic and professional characteristics of the healthcare workers.

To identify independent predictors of hand hygiene beliefs and practices, a multivariable analysis was performed using Generalized Linear Models (GLM). Two separate GLM models were constructed with Hand Hygiene Belief (HHBS) and Hand Hygiene Practice (HHPI) scores as the dependent variables. All sociodemographic and professional variables were included as independent variables in the models. Categorical variables were entered into the models using reference categories to determine the relative effect size of each group. Statistical significance was defined as a P-value of less than 0.05 with 95% confidence intervals (CIs).

### Ethics considerations

This study was approved by the Clinical Research Ethics Committee of Recep Tayyip Erdoğan Training and Research Hospital in Turkey on March 30, 2024 (reference number: MSTH/17691). The researchers explained the study’s aims to the healthcare workers and requested their participation. Each participant provided written informed consent after receiving the complete information. Only those who willingly consented to participate were included in this study. After obtaining informed consent, participants completed the questionnaires independently. Participants were instructed to submit the completed questionnaires privately to the principal researcher. Additionally, we provided participants with pertinent information regarding their right to discontinue their involvement in the study at any time. Furthermore, we ensured that the questionnaires were conducted with the promise of anonymity, adhered to stringent confidentiality measures, and refrained from revealing identifiable personal information. All methods were performed in accordance with the relevant guidelines and regulations.

## Result

### Demographics characteristics of healthcare workers

A total of 304 healthcare workers participated in this study, achieving our target sample size. Of the hospital’s 1,180 HCWs, 304 were randomly selected and invited to join via stratified random sampling. All 304 selected participants agreed to enrol and completed usable questionnaires, yielding a 100% response rate among those invited (304/304 = 100%). This represents 25.8% of the facility’s total HCW population. Most participants were between 25 and 30 years old (37.5%), with males comprising 53.6%. Most healthcare workers held a bachelor’s degree (75.3%), with nurses constituting the largest professional group (45.4%). The majority of participants had 6–10 years of experience (40.5%), and most worked in inpatient departments (34.5%). (Table 1**)**.

**Table 1:** Sociodemographic and professional characteristics of participants (*N* = 304).

Variable	Category	Frequency (*n*)	Percentage (%)
**Age Group**	< 25 years	72	23.7
	25–30 years	114	37.5
	30–35 years	72	23.7
	35 + years	46	15.1
**Gender**	Male	163	53.6
	Female	141	46.4
**Marital Status**	Married	139	45.7
	Unmarried	165	54.3
**Education Level**	Diploma	31	10.2
	Bachelor’s Degree	229	75.3
	Master’s Degree	44	14.5
**Profession**	Doctor	62	20.4
	Nurse	138	45.4
	Midwife	36	11.8
	Pharmacist	17	5.6
	Allied Health	22	7.2
	Others	29	9.5
**Work Experience**	< 1 year	62	20.4
	1–5 years	70	23.0
	6–10 years	123	40.5
	10–15 years	30	9.9
	> 15 years	19	6.3
**Department**	Inpatient (Wards)	105	34.5
	Outpatient	42	13.8
	Laboratory	18	5.9
	OT (Surgery)	50	16.5
	Emergency	33	10.9
	ICU	56	18.4
**Total**		**304**	**100.0**

### Hand hygiene beliefs scale (HHBS)

The study found that hand hygiene is a critical aspect of clinical practice, with 61.2% of the respondents stating that it is essential. Professional accountability was also evident, with 49.3% acknowledging their roles as role models. The study also revealed that hand hygiene reduced patient mortality and infection-associated costs. However, 60.5% of respondents admitted that remembering to practice hand hygiene requires conscious effort, and implementation barriers were identified. Additionally, challenges in professional dynamics were noted, with only 31.9% feeling empowered to challenge incorrect practice (Table 2**).**

**Table 2:** Beliefs regarding hand hygiene among healthcare workers (*N* = 304).

Hand Hygiene Beliefs Scale (HHBS)	SD (%)	DA (%)	*N* (%)	A (%)	SA (%)
Hand hygiene training is considered an important part of the curriculum	1.3	3	1.6	32.9	61.2
The clinical services I practice emphasize the significance of hand hygiene, making it easier to emphasize.	2	9.2	4.9	39.5	44.4
My clinical consultant/service manager emphasizes the importance of hand hygiene	3.6	18.1	9.2	46.4	22.7
I must be a role model for other healthcare professionals	0.7	7.2	5.6	49.3	37.2
When I am busy, completing my work is more important than paying attention to hand hygiene.	29.3	58.9	5.6	5.6	0.7
Performing hand hygiene in recommended situations can reduce the patient mortality rate.	1	14.8	3.3	60.2	20.7
Performing hand hygiene is recommended in cases can reduce the costs associated with infections	0	2	4.6	53	40.5
Since my patients’ needs are a priority, in the recommended cases I cannot always perform hand hygiene	2.6	31.6	7.2	44.1	14.5
Preventing nosocomial infections is an important part of the role of healthcare professionals	0.7	5.3	8.6	36.2	49.3
Experienced in performing or not performing hand hygiene I take the behavior of healthcare professionals as an example	2.6	10.9	9.2	56.9	20.4
Getting healthcare-associated infections could threaten my life or career.	2	6.6	19.1	45.7	26.6
I believe that I have the power to change wrong/bad practices in the work environment.	7.6	26	7.6	31.9	27
Failure to perform hand hygiene in recommended situations is considered negligence conceivable	1.3	56.6	2	26.3	13.8
Hand hygiene is a habit for me in my personal life	0	2.6	4.3	54.3	38.8
I can use my knowledge of hand hygiene effectively in my clinical work; I am confident that I can implement it	0	1	4.3	62.2	32.6
Remembering to perform hand hygiene in recommended situations requires effort	0	6.3	6.3	60.5	27
It would bother me to warn a healthcare professional about hand washing	3	13.2	13.2	52	18.8
Providing hand hygiene slows down the build-up of immunity against diseases.	0.3	17.8	17.1	45.4	19.4
Dirty sinks may be a reason not to wash hands	11.5	34.2	14.5	19.1	20.7
Lack of a suitable cleaning product can be a reason for not cleaning hands	1.6	23	7.2	46.7	21.4
Providing hand hygiene after caring for a wound can protect against infections	0.7	17.8	4.6	29.3	47.7
Cleaning hands after using the toilet reduces the risk of infectious disease transmission	0	0	0	48	52

SD: Strongly Disagree DA: Disagree N: Neutral A: Agree DA: Disagree.

### Hand hygiene practices inventory (HHPI)

Observational data on hand hygiene practices in clinical settings revealed varying levels of compliance across different scenarios. The highest adherence rate was observed for isolation protocols, with 64.8% of healthcare professionals consistently performing hand hygiene before entering an isolated patient’s room. In routine patient care, 57.2% of patients reported intermittent hand hygiene after using the toilet, whereas 53% practiced intermittent hand hygiene before wound care. Post-procedure hygiene showed that 60.2% of the patients intermittently performed hand hygiene after wound care. Potential patient safety concerns arose regarding invasive procedures, with 67.1% of healthcare providers intermittently performing hand hygiene before inserting an invasive instrument and 49.7% after the procedure. Compliance is low for contact with bodily fluids, as 66.1% of healthcare workers intermittently perform hand hygiene after exposure. There is room for improvement in personal protective equipment protocols, with only 35.2% consistently performing hand hygiene after removing gloves. In the patients’ contact scenarios, 44.1% performed hand hygiene tasks intermittently, while 25.7% did so consistently. For visibly soiled hands, 28.6% consistently performed hand hygiene; in specific clinical procedures, only 24.7% consistently adhered to hand hygiene practices (Table 3**)**.

**Table 3:** Hand hygiene practices inventory among healthcare workers in clinical settings (*N* = 304).

Hand hygiene practices inventory (HHPI)	Never (%)	Sometimes (%)	Often (%)	Most of the time (%)	Always (%)
I perform hand hygiene in the hospital in the following situations: After going to the toilet	0.3	57.2	5.6	16.4	20.4
Before caring for the wound	2.3	53	19.1	25.7	0
After caring for the wound	0.3	60.2	0.3	30.6	8.6
After touching possibly contaminated objects	1	43.1	9.9	30.9	15.1
After contact with blood and body fluids;	6.6	66.1	6.9	18.4	2
Before inserting an invasive instrument into a patient	0	67.1	0.3	31.9	0.7
After inserting an invasive instrument into a patient	0.3	49.7	4.6	43.8	1.6
Before entering the room of the isolated patient	0	20.7	12.5	2	64.8
After touching the patient’s skin	3.3	44.1	5.6	21.4	25.7
After entering the room of the isolated patient	2.3	18.8	8.9	50	20.1
Before endotracheal aspiration	11.5	21.7	21.7	20.4	24.7
After contact with patients’ secretions	20.4	11.2	38.2	11.2	19.1
After the patient’s contacts	5.6	37.2	11.2	24.7	21.4
After taking off the gloves	10.9	30.6	0	23.4	35.2
When my hands feel or look dirty	6.9	35.2	4.9	24.3	28.6

### Factors associated with hand hygiene beliefs

The multivariable analysis using a Generalized Linear Model (GLM) indicated that education, profession, and years of experience were significant predictors of hand hygiene belief scores. Specifically, healthcare workers with a Master’s degree were associated with significantly lower belief scores than the reference group (β = −0.257, *p* = 0.041). In terms of professional roles, being a pharmacist was significantly and positively associated with higher belief levels (β = 0.310, *p* = 0.032). Furthermore, professional experience significantly influenced beliefs, as healthcare workers with 10–15 years of experience showed a substantial decrease in belief scores compared with those with less than 1 year of experience (β = −0.390, *p* = 0.001). (Table 4**)**.

**Table 4:** Generalized linear model (GLM) analysis of factors associated with hand hygiene Beliefs.

Variable	Category	Coefficient ($\:\boldsymbol{\beta\:}$)	Std. Error	z-value	*p*-value	[95% Conf. Interval]
**Age Group**	< 25	—	—	—	(Ref.)	—
	25–30	0.043	0.077	0.56	0.579	[−0.109, 0.195]
	30–35	0.102	0.090	1.13	0.260	[−0.075, 0.279]
	35+	0.043	0.103	0.42	0.674	[−0.159, 0.246]
**Gender**	Male	—	—	—	(Ref.)	—
	Female	−0.048	0.058	−0.83	0.408	[−0.162, 0.066]
**Marital Status**	Married	—	—	—	(Ref.)	—
	Unmarried	−0.022	0.061	−0.37	0.709	[−0.142, 0.097]
**Education**	Diploma/Other	—	—	—	(Ref.)	—
	Bachelor’s Degree	−0.186	0.104	−1.78	0.074	[−0.391, 0.018]
	Master’s Degree	−0.257	0.126	−2.04	0.041*	[−0.504, −0.010]
**Profession**	Doctors	—	—	—	(Ref.)	—
	Nurse	0.119	0.081	1.46	0.144	[−0.040, 0.278]
	Midwife	0.096	0.109	0.88	0.378	[−0.117, 0.310]
	Pharmacist	0.310	0.144	2.15	0.032*	[0.027, 0.594]
	Allied Health	0.178	0.134	1.33	0.184	[−0.085, 0.442]
	Others	0.026	0.122	0.21	0.831	[−0.214, 0.266]
**Experience**	< 1 Year	—	—	—	(Ref.)	—
	1–5 Years	−0.139	0.089	−1.56	0.120	[−0.315, 0.036]
	6–10 Years	0.025	0.080	0.32	0.748	[−0.132, 0.183]
	10–15 Years	−0.390	0.114	−3.40	0.001*	[−0.616, −0.165]
	> 15 Years	0.169	0.131	1.29	0.196	[−0.087, 0.426]
**Work Shift**	Day	—	—	—	(Ref.)	—
	Night	0.051	0.076	0.67	0.504	[−0.099, 0.201]
	Both (Rotation)	−0.086	0.068	−1.26	0.208	[−0.221, 0.048]
**Department**	Inpatient/Wards	—	—	—	(Ref.)	—
	Outpatient	−0.151	0.098	−1.54	0.122	[−0.343, 0.040]
	Laboratory	−0.055	0.127	−0.44	0.663	[−0.305, 0.194]
	OT	0.068	0.087	0.77	0.439	[−0.104, 0.240]
	Emergency	−0.038	0.099	−0.38	0.701	[−0.234, 0.157]
	ICU	−0.061	0.083	−0.74	0.461	[−0.226, 0.102]

(Ref.) = Reference category; * = Statistically significant at p < 0.05. .

### Factors associated with hand hygiene practice

The GLM identified several significant demographics, educational, and professional determinants of hand hygiene practice. Regarding demographic characteristics, female gender was significantly associated with higher practice scores than male gender (β = 0.981, *p* = 0.034), while being unmarried was associated with a significant decrease in practice scores (β = −1.250, *p* = 0.010). Educational attainment emerged as a strong positive predictor, with both Bachelor’s degree holders (β = 2.115, *p* = 0.011) and Master’s degree holders (β = 2.538, *p* = 0.011) demonstrating significantly higher practice scores than the reference group. Professionally, nurses were found to have considerably lower practice scores than doctors (β = −1.659, *p* = 0.010). Conversely, having 10–15 years of experience was associated with a significant increase in hand hygiene practice scores (β = 2.444, *p* = 0.007). Finally, the hospital department was a substantial factor, as staff in the laboratory exhibited significantly higher practice scores than those in the reference department (β = 3.090, *p* = 0.002). (Table 5**)**.

**Table 5:** Generalized linear model (GLM) analysis of factors associated with hand hygiene Practice.

Variable	Category	Coefficient ($\:\boldsymbol{\beta\:}$)	Std. Error	z-value	*p*-value	[95% Conf. Interval]
**Age Group**	< 25	—	—	—	(Ref.)	—
	25–30	−0.681	0.617	−1.10	0.270	[−1.891, 0.529]
	30–35	−1.406	0.720	−1.95	0.051	[−2.817, 0.005]
	35+	−1.031	0.825	−1.25	0.211	[−2.647, 0.586]
**Gender**	Male	—	—	—	(Ref.)	—
	Female	0.981	0.464	2.11	0.034*	[0.072, 1.890]
**Marital Status**	Married	—	—	—	(Ref.)	—
	Unmarried	−1.250	0.487	−2.57	0.010*	[−2.206, −0.295]
**Education**	Diploma/Other	—	—	—	(Ref.)	—
	Bachelor’s Degree	2.115	0.832	2.54	0.011*	[0.484, 3.745]
	Master’s Degree	2.538	1.003	2.53	0.011*	[0.573, 4.504]
**Profession**	Doctors	—	—	—	(Ref.)	—
	Nurse	−1.659	0.648	−2.56	0.010*	[−2.930, −0.389]
	Midwife	−0.362	0.869	−0.42	0.677	[−2.065, 1.341]
	Pharmacist	−0.366	1.152	−0.32	0.750	[−2.624, 1.891]
	Allied Health	−1.320	1.071	−1.23	0.218	[−3.419, 0.779]
	Others	0.011	0.975	0.01	0.991	[−1.899, 1.922]
**Experience**	< 1 Year	—	—	—	(Ref.)	—
	1–5 Years	0.629	0.714	0.88	0.378	[−0.770, 2.029]
	6–10 Years	1.149	0.641	1.79	0.073	[−0.107, 2.405]
	10–15 Years	2.444	0.913	2.68	0.007*	[0.654, 4.233]
	> 15 Years	1.140	1.043	1.09	0.274	[−0.904, 3.183]
**Work Shift**	Day	—	—	—	(Ref.)	—
	Night	0.283	0.611	0.46	0.643	[−0.914, 1.479]
	Both (Rotation)	0.629	0.547	1.15	0.250	[−0.443, 1.701]
**Department**	Inpatient/Wards	—	—	—	(Ref.)	—
	Outpatient	0.880	0.780	1.13	0.259	[−0.648, 2.408]
	Laboratory	3.090	1.013	3.05	0.002*	[1.105, 5.075]
	OT	0.887	0.699	1.27	0.204	[−0.482, 2.256]
	Emergency	1.242	0.795	1.56	0.118	[−0.316, 2.799]
	ICU	1.159	0.667	1.74	0.082	[−0.147, 2.466]

(Ref.) = Reference category; * = Statistically significant at p < 0.05. .

## Discussion

This study examined hand hygiene beliefs and practices among healthcare workers (HCWs) at a tertiary hospital in Mogadishu, Somalia. Regarding hand hygiene beliefs, our findings revealed a relatively high overall score, indicating that HCWs generally recognize the importance of hand hygiene in preventing healthcare-associated infections. These findings are consistent with those of the studies conducted in Ethiopia^20^ and India^2^. This disparity is frequently observed in healthcare settings, particularly in resource-limited environments^4,5^. Factors beyond belief systems influence hand hygiene adherence, including infrastructural limitations, time constraints, and workload pressures, which are commonly found in resource-limited healthcare environments^7,8^.

Level of education had a notable impact on belief scores, with individuals holding master’s degrees showing the highest average scores, followed by those with bachelor’s degrees or diplomas. The positive association between education and hand hygiene beliefs is consistent with previous studies, indicating that higher education contributes to a deeper understanding of infection prevention protocols^2,9^. The higher belief scores among those with advanced degrees may reflect greater exposure to infection prevention guidelines and more frequent participation in continuing professional development, rather than the degree itself being the determining factor. This interpretation highlights that practical, context-specific training interventions are likely to be more impactful than educational qualifications alone in improving hand hygiene practices.

Significant differences in attitude scores were also observed across professional roles, with doctors scoring highest and pharmacists lowest. A comparable study supports this finding^7^. This discrepancy may be attributed to variations in training and emphasis placed on infection control in direct patient care roles across different healthcare professions^20^.

Years of experience significantly influenced belief scores among healthcare workers (HCWs), with those with 10–15 years of experience demonstrating the highest average scores. This observation aligns with the findings of previous studies^4,20^. HCWs with extensive experience may have accumulated substantial knowledge and skills while adhering strictly to established protocols, leading to higher confidence in hand hygiene practices.

Work shifts significantly impacted employee attitude scores, with individuals working both day and night shifts having the highest ratings. This outcome aligns with previous research findings^8^. The range of supervisory methods and learning opportunities available across various work shifts may explain the differences in scores.

The practice scores were notably influenced by educational attainment, with those holding master’s degrees exhibiting the highest level of compliance. This observation aligns with a similar study^13^. A possible explanation for this trend is that individuals with advanced education are more likely to understand proper procedures, receive specialized training, and exhibit higher levels of responsibility and professionalism, resulting in improved compliance rates.

A significant correlation was observed between gender and practice scores, with female healthcare workers (HCWs) demonstrating superior compliance compared with their male counterparts. This outcome is consistent with findings from earlier studies^7,21,22^. The observed difference in compliance may be attributed to the tendency of female HCWs to pay closer attention to detail, to be more risk-aware, and to adhere more strictly to protocols. Additionally, cultural and social factors may contribute to more rigorous adherence and, consequently, higher practice scores.

Significant differences in practice scores were observed across departments, with laboratory personnel demonstrating the highest level of compliance and inpatient staff at the lowest level. This observation aligns with the findings of the current study^21^. This may be attributed to the laboratory staff’s strict adherence to established protocols, which yields superior compliance rates. By contrast, inpatient staff often face fluid situations, time pressures, and heavy workloads, which could contribute to their lower compliance levels.

Compliance scores were notably linked to experience levels, with healthcare workers who had 10–15 years of experience in the field exhibiting the highest adherence to practices. This finding is consistent with those of previous studies^23,24^. This phenomenon may be attributed to increased work experience and the heightened likelihood of receiving training, assuming greater responsibility, and developing a stronger sense of organizational affiliation.

This study has several strengths. First, stratified random sampling was employed to ensure proportional representation and minimize selection bias, providing a comprehensive understanding of hand hygiene practices. Second, diverse professional roles offer a comprehensive view of hand hygiene practices across the healthcare system, facilitating more targeted interventions. However, limitations include the potential introduction of social desirability bias through self-reported data, the cross-sectional nature that restricts causal relationships, and the study’s limited applicability to other healthcare settings in Somalia or low-resource environments due to differences in resources, protocols, and organizational cultures.

## Conclusion

This study reveals a significant and concerning gap between healthcare workers’ strong beliefs in the importance of hand hygiene and their moderate, inconsistent application in clinical practice. While knowledge is high, it does not translate into reliable behavior, with remarkably low compliance in critical situations such as after glove removal and during invasive procedures. Key demographic factors, including education level, experience, and department, significantly influence compliance, indicating that a uniform approach to improvement is inadequate. The findings underscore that enhancing patient safety requires moving beyond awareness campaigns to implement targeted, systemic interventions. Therefore, concrete actions such as establishing peer-mentoring programs for new staff, implementing mandatory refresher training for senior professionals, and introducing consistent observational monitoring with direct feedback are essential to bridge the critical divide between knowing and doing.

## Data Availability

The data used in this study shall be available upon reasonable request from the corresponding author.
